# Using Social Networks and Model Simulations of Social Disruption to Identify Alternative Translocation Strategies for the Endangered Cooperative-Breeding Floreana Mockingbird

**DOI:** 10.3390/biology15120912

**Published:** 2026-06-10

**Authors:** Enzo M. R. Reyes, Adam N. H. Smith, Christian Sevilla, Michelle M. Roper, Dianne H. Brunton

**Affiliations:** 1 Ecology and Conservation Lab, School of Natural and Computational Sciences, Massey University, Private Bag 102-904 North Shore Mail Centre, Auckland 0632, New Zealand; 2 Sea Through Science Limited, Auckland 0932, New Zealand; 3 Dirección de Ecosistemas, Dirección del Parque Nacional Galápagos, Av. Charles Darwin, S/N, Puerto Ayora 200102, Islas Galápagos, Ecuador; 4 School of Biological Sciences—Te kura Mātauranga Koiora, University of Auckland—Waipapa Taumata Rau, Auckland 1142, New Zealand

**Keywords:** social networks, Floreana Mockingbird, reintroduction, exponential random graph models, Galapagos, conservation, cooperative breeding

## Abstract

(1) The importance of social structure and dominance hierarchies in cooperative-breeding species is well-documented, yet the inclusion of these processes in conservation translocation planning remains limited. Here, we empirically measured the social networks of three populations of the endangered Floreana Mockingbird, then used model simulations of different translocation scenarios to test the effects of social disruption on the social networks. (2) We used social network analysis and Exponential Random Graph Models (ERGMs) to characterise dominance hierarchies, group structure, and the consequences of selectively removing individuals from family groups. (3) Dominance hierarchies were strongly transitive, with age emerging as the primary determinant of dominance relationships. Simulated removals demonstrated that the loss of individuals occupying different network positions produced variable levels of social disruption. (4) Although age is the principal driver of antagonistic interactions, network properties such as high betweenness centrality and the presence of a broker (individuals that occupy a strategic position) are also critical considerations for translocation design. Incorporating social network structure into management strategies can minimise group disruption and enhance the success of conservation translocations for endangered cooperative breeders.

## Simple Summary

In this study, we examined how social structure and dominance hierarchies could influence conservation translocations in the endangered Floreana Mockingbird. We analysed social networks across three family groups and used network simulations to assess how removing individuals affects group dynamics. Our results showed that dominance hierarchies are strongly structured, with age as the main determinant of dominance. However, the removal of individuals from different network positions led to varying levels of social disruption. We found that individuals occupying key roles, such as those with high connectivity or acting as brokers, are particularly important for maintaining social stability. Overall, our findings indicate that considering only traits like age could be insufficient, as the position of individuals within the social network is also critical. Incorporating these social network insights into translocation planning can help minimise disruption and improve conservation outcomes for cooperative breeding species.

## 1. Introduction

Cooperative breeding is a mating system in which some component of parental care is shared between parents and helpers [1,2,3]. Helpers are typically genetically related juveniles or sexually mature birds that are reproductively suppressed due to limited breeding opportunities or low reproductive rates within the family group [4]. In birds, cooperative breeding systems have been observed in over 800 species, and the varied roles of these helpers can include direct food provisioning of chicks, territorial defence, and sharing incubation and brooding duties [5,6,7,8].

Cooperative breeding birds exhibit complex social attributes, including dominance structures and hierarchies, that strongly affect demographic parameters such as population growth rate, sex ratio, and effective population size. These parameters are crucial for conservation management planning aimed at sustaining or increasing population viability [9]. The viability of a wild population is strongly associated with social interactions among its members, and events that fragment social interactions can have long-term consequences at both individual and population levels [10]. For example, the loss of high-ranking individuals (through mortality or dispersal) could collapse or disrupt an entire social group [11,12,13,14].

Similar to mortality and dispersal events, conservation actions like translocations can disrupt social interactions in social species, potentially influencing the success of these actions. In previous translocations of cooperative breeders, translocated birds typically form breeding pairs without helpers shortly after release [15,16,17,18]. Additionally, there is evidence that the disruption of social interactions in early life stages could affect the reproductive success and influence the survival of adult individuals. For instance, in the Spotted hyena (*Crocuta crocuta*), the annual reproductive success and longevity of individuals are positively related to the number of social interactions during early life stages [19]. Similarly, Orange-bellied Parrots (*Neophema chrysogaster*) that form social ties during the juvenile stage are more likely to survive their first migration compared with captive-reared birds that had not had the opportunity to socialise before their release [20]. There is some evidence in birds and mammals that moving entire social groups minimises this risk and reduces group disintegration, thereby improving fidelity at the translocation sites [21,22,23]. In the context of conservation translocations, social interactions have been used mostly during the post-release phases to investigate the interaction between individuals and the spread of diseases [24,25,26].

Social network analysis (SNA) is a tool used to investigate associations and interactions among individuals. SNA is a framework that quantifies social structure at multiple levels, such as individuals, groups or populations [27]. In SNA, individuals within a population are represented as connected *nodes*, with associations or interactions between two individuals represented by *ties* [28]. *Centrality* and *density* (Box 1) are well-known basic measures of the connectivity properties of social networks. While informative, these measures are insufficient to capture the mechanisms driving complex social behaviour, particularly when network structure arises from multiple independent mechanisms. For example, patterns of clustering, reciprocity or homophily may emerge simultaneously from self-organisation, structural balance, and node-level attributes, making it difficult to disentangle their relative contributions using individual network measures in isolation [29]. Although a wide range of traditional network metrics can describe specific structural features, they do not explicitly model how these features arise jointly.

Exponential random graph models (ERGMs) address this limitation by allowing multiple structural and attribute-based effects to be modelled simultaneously within a unified probabilistic framework, thereby linking observed social network structure to underlying processes that generate social interaction [30]. ERGMs allow the modelling of multiple variables at the same time, account for directed and weighted interactions between nodes (when interactions have a clear direction from sender to receiver and multiple times), and allow the evaluation of the model through simulations [31,32,33]. ERGMs have been used to model dominance interactions and hierarchies within family groups in cooperative breeding species [34,35]. They have also been used as indirect evidence of social cohesion [36].

Box 1 **Social network analysis terminology used in this article. Terms definitions are based on ref. [37].**

**General network glossary**

**Nodes/vertices:** Basic elements of a network, nodes represent individuals in the network.

**Ties/edges:** Basic elements of a network, ties represent interaction between nodes.

**Degree:** Is the number of ties connected to a given node.

**Centrality:** Determined by the shortest path and geodesic, it is defined as the average of all the paths that a node will take to reach the rest of the network.

**Betweenness centrality:** Defined as the number of times a node lies in the shortest path of the other network nodes.

**Transitivity:** Determines the number of triangles of which a node is part.

**Density:** Defined as the proportion of observed ties in a network to the maximum number of possible ties.

**Directed network:** When ties in the network have a direction from a sender to a receiver.

**Undirected network:** When ties in the network do not have a specific direction between them, but are still connected.

**Weighted networks:** Networks where the ties have a value as the number of interactions between nodes.

**Components of a network:** Defined as sections of the network in which nodes are internally connected but not tied to other sections of the network.

**Isolates components of a network:** A section of the network where the nodes have been disconnected from other components. An isolate is defined as a node with 0 degree.

One of the key objectives of SNA is to quantify the position of an individual in the social network structure; this tool can be used to inform changes in social structure that are not detectable using traditional methods, such as population size or dyadic relationships [38]. Despite being powerful tools in behavioural studies, the usefulness of SNA and ERGM in the field of conservation biology is not fully developed. Nonetheless, this type of analysis could likely be applied to minimise social group dissolutions caused by translocations, by identifying key individuals that are essential for maintaining social stability [39], thereby facilitating rapid establishment in a new area. The potential value of these approaches is especially evident in systems with complex social organisation, such as the Galapagos Mockingbirds [40,41].

All four species of Galápagos Mockingbirds have distinct phenotypes and are allopatric, with three species living on different single islands (*Mimus melanotis* on San Cristobal Island, *M. macdonaldi* on Española Island, and *M. trifasciatus* restricted to two small islets near Floreana Island). Only *M. parvulus* is widespread throughout the archipelago [40]. Mockingbirds in the Galápagos display a wide range of social organisations depending on the species and location, with limited availability of habitat reported to be the main driver of cooperative breeding in all four species [41]. The social structures of each species range from *M. melanotis* with socially monogamous pairs or trios without helpers [41] to *M. macdonaldi*, which has extensive family groups with helpers [42]. In addition, social structures range from singular cooperative breeding, in which only the dominant pair breeds, to plural cooperative breeding, where more than one pair breeds within the boundaries of a family group territory. These two social structures have been recorded for *M. parvulus*, *M. trifasciatus* and *M. macdonaldi* [6,42].

Although social structures have been well studied in three of the four Galápagos Mockingbird species, the sociality of the Floreana Mockingbird (*M. trisfasciatus*) has only been partially researched in one of its two populations. These descriptions are limited to the small population on Champion Islet, between 20 and 50 individuals constrained by limited habitat [43]. On Champion Islet, Floreana Mockingbirds have been reported as an obligate cooperative breeder, with groups composed of a dominant male and female, and up to four submissive birds or helpers [44]. Therefore, they are categorised as mostly singular breeders but with rare cases of plural breeding [41,45].

We investigated the social structures of the endangered Floreana Mockingbird, a species proposed for future conservation translocations, on two islands and modelled the effects of hypothetical social disruptions associated with translocations from source populations. We focused on dominance interactions, a key feature of cooperative breeding groups, and proposed the following hypotheses: (a) Larger individuals dominate smaller ones; (b) older birds have higher social status and dominate younger individuals, reflecting age-based homophily; (c) dominance interactions occur more frequently between individuals of the same sex (sexual homophily); (d) dominance interactions show reciprocity (by reciprocity, we refer to whether an aggressive interaction directed from individual *A* to individual *B* increases the probability of a corresponding aggressive interaction from *B* back to *A*), with a higher likelihood of mutual interactions. We applied social network analysis (SNA) to field observations collected from three family groups to: (1) characterise dominance network structure in both extant populations, (2) examine the influence of group and individual attributes, and (3) assess potential social disruption by modelling the removal of key individuals under different reintroduction scenarios.

## 2. Materials and Methods

### 2.1. Study Area and Field Methods

This study took place during July/August 2019 on the islets of Champion (90°23′100″ W 01°14′240″ S) and Gardner-by-Floreana (hereafter Gardner) (90°17′700″ W 01°19′969″ S), located in the northeast and southeast of Floreana Island, Galápagos Archipelago (for details, see ref. [46] and Supplementary Material). We selected three different social groups of different sizes, and all birds were banded with unique colour combinations added to existing metal ID bands for individual recognition. During banding, morphological measurements were taken, and age was assessed [47]. Juveniles were identified based on their distinct plumage, and adults were aged based on the year of banding (from 2006 onwards). Finally, sex was determined by wing chord measurements using methods described in [46].

### 2.2. Dominance Observations

Behavioural observations were conducted during the non-breeding season on two family groups on Gardner Islet and one group on Champion Islet. Observations were conducted from 6:30 to 17:30 h (Galapagos local time). Focal groups were observed for 60 min in the morning and 60 min in the afternoon over 12 days of observations (a total of 24/person hours). Behaviour was observed in a shrubby habitat with open spaces of low vegetation types, so the observers had a full view of the birds. Observers walked into the focal family group territories opportunistically recording interactions between birds.

Territory boundaries were inferred from the GPS position of the birds during the first few days of banding and re-sighting. Interactions between individuals were recorded using a 50–300 mm Nikon (Tokyo, Japan) lens and Eagle Optics Ranger 10 × 42 binoculars (Yiwu, China). Dominance was recorded during two forms of interactions within family groups: aggressiveness, such as pecks, chasing [48] and dominance postures, and submission, such as a crouched posture [48]. These observations were only recorded between dyads. The identities of senders and receivers of the interactions were recorded. Interactions between members of the focal group and other family groups were not collected due to time constraints. Floreana Mockingbirds are habituated to the presence of researchers, but to minimise any disturbance of normal activities on the birds, the observer arrived 10 min prior to observations for acclimatation. Mockingbirds are curious but usually lose interest in humans quickly.

### 2.3. Statistical Analysis

#### 2.3.1. Dominance Hierarchy Structure

A glossary of the general terms of social network analysis used in this paper can be found in Box 1. Box 2 shows the terminology of the ERGM used in our analysis. We created a directed binary matrix of dominance interactions (aggressiveness and submission pooled as there is a directional dominance relationship) for each family group [49]. Birds were represented by *nodes,* and the presence (1) or absence (0) of interactions was indicated by the *edges* of the network. To create directionality, the *edges* of each matrix were arranged by senders as rows and receivers as columns. To test for the dominance hierarchy structure of the Floreana Mockingbird, we used the triangle transitivity method of Shizuka and McDonald [50], which is equivalent to linear regression in traditional linearity measures of dominance hierarchies [51]. Transitivity was calculated separately for each family group. This method measures the proportion of groups that form transitive triads (*t_tri_*) (e.g., individual *A* dominates *B* and *B* dominates *C*, then *A* also dominates *C*) as opposed to cyclical triads [52]. Finally, *t_tri_* and its statistical significance were calculated in the *statnet* package in R (Version 2023.06.1+524) [53] following the code of Shizuka and MacDonald [50] and Corrigendum. The combined p-values were calculated using Fisher’s combined probability test, which combines the three group-specific *p*-values into a single overall test statistic [54] in R [55].

#### 2.3.2. Dominance Network Structure

We created a global matrix containing the three groups to identify the structure of our network. The matrix used was directed and weighted for this analysis, which means that the exact number of interactions between dyads was recorded and specified as an *edge* attribute of the network. For the body size attribute, we used the Scaled Mass Index (SMI) of the mass and the head-bill morphometry following Equation (1) described in ref. [56].

(1) $$\hat{\;M_{i}}=M_{i}\;{\left[\frac{L_{0}}{L_{i}}\right]}^{b_{SMA}}$$

where $M_{i}$ and $L_{i}$ are the body mass and the linear body measurement of individual i respectively; $b_{SMA}$ is the scaling exponent estimated by the SMI regression of M on L; $L_{0}$ is the arithmetic mean value for the study population; and $\hat{M_{i}}$ is the predicted body mass for individual i when the linear body measure is standardised to $L_{0}$. We performed a Poisson distribution ERGM with the following predictor variables in our model (Box 2): *sum*, *non_zero, node_factor*, *node_match*, *abs_diff*, *node_ocov*, *node_icov* and *mutual*.

Box 2 **ERGM statistical terms applied in this paper. Terms definitions are based on ref. [37].**

**Statistical ERGM terms applied to our model**

**Sum:** Analogous to the intercept in a regression model

**Non_zero:** Defined as the count of ties with a value above zero.

**Node_factor:** Defined as the actor effect of sex that tests the likelihood of one sex initiating dominance interactions.

**Node_match:** For sex and age, examines if interactions are more likely between same-sex pairs and same-age pairs.

**Abs_diff:** For SMI, which examines the difference in size.

**node_ocov:** Actor for age that tests if age influences the likelihood of initiating dominance interactions.

**Node_icov:** Receiver for age that tests if age influences the likelihood of receiving dominance interactions.

**Mutual:** For interaction reciprocity.

Additionally, we used the *offset* argument in the model to restrict interactions within the studied groups. More details regarding the statistical terms of the model can be found in ref. [57]. Additionally, we tested for model degeneracy (unrealistic model behaviour or poor fit to data) using Markov Chain Monte Carlo (MCMC) diagnosis in the package ERGM. For more details about model degeneracy, see ref. [58]. Finally, we evaluated the resemblance of the mean of the model-simulated networks (generated from the fitted ERGM) using the statistics of the observed network.

### 2.4. Translocation Simulation

On the directed and weighted network, we calculated the *betweenness-centrality* using the *statnet* package [53] in R [55]. *Betweenness-centrality* of a *node* is defined as the number of shortest paths in which a *node* lies within the network [59,60], i.e., an individual with high betweenness acts as a bridge between the different parts of the network. Further, we identified *cut-points* (*brokers*) using the inbuilt function in *statnet.* Brokers are *nodes* that, when removed, affect the flow and connectivity properties of the network [61,62]. Although related, these metrics capture different aspects of network structure. Betweenness centrality identifies individuals that connect many other individuals within the network, whereas brokers (cut-points) are individuals whose removal would disconnect parts of the network. Thus, high-betweenness individuals are socially central, while brokers are structurally critical. We simulated ‘removal for translocation scenarios’ by removing individuals from the network. Our simulation of removal consisted of relocating up to 50% of individuals from each family group, which is intended to represent a potentially realistic translocation situation in which managers aim to capture and move an entire family group but are unable to capture all individuals. We simulated five scenarios with different *node* removals. (1) Age: older birds (>1 year) and (2) juveniles (<1 year old) were removed. (3) Node: individuals with high *betweenness* were removed. (4) Brokers: Brokers of each group were removed. (5) Random removal of 50% of individuals within each family. To assess the impact of the “removal” of *nodes,* we used two network metrics: the number of network components and the number of isolated nodes, which measure the number of subgroups and isolated individuals separated from the main networks. Metric values were obtained using the *statnet* package. Random selection of individuals and network plots of the different scenarios were performed in R [55].

## 3. Results

Descriptive statistics are presented as a general summary of the dataset. We observed a total of 37 individuals from three different groups. We recorded a total of 137 dominance interactions, ranging from 1 to 16 per individual and an average of 4.9 (±0.8 SE) interactions per individual.

### 3.1. Hierarchy and Network Structure

**Figure 1:**
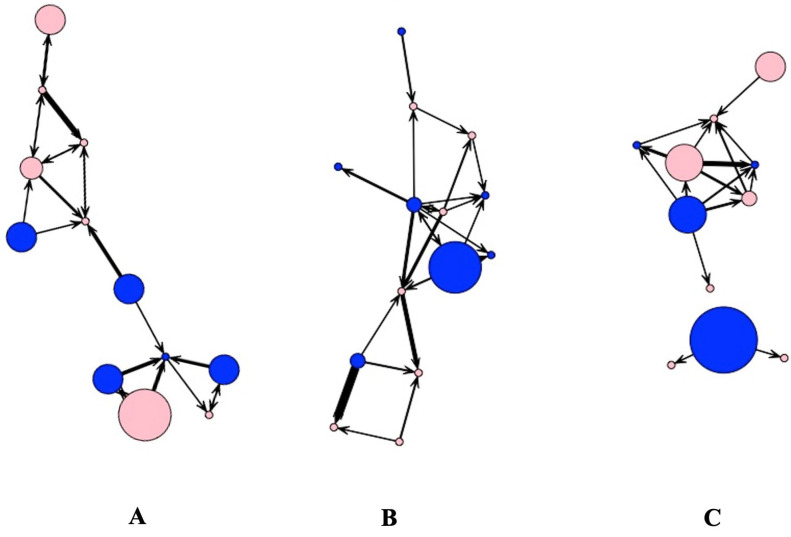
Networks of dominance displays of three Floreana Mockingbird family groups (groups (**A**,**B**) from the Gardner population and group (**C**) from the Champion population). Nodes represent individual birds. Networks are displayed by sex (blue nodes represent males and pink nodes represent females) and age (node size was scaled to the age of the individuals), where large nodes represent older birds, and smaller nodes represent young birds. Tie thickness represents the number of interactions between individuals (nodes) and arrows represent the directionality of the interaction: aggressor (actor) > target (receiver). Figures were created using the *statnet* package.

Dominance networks in the Floreana Mockingbirds did not contain cyclic triads (*P_t_* = 0.93; *t_tri_* = 0.73). Furthermore, this result was not significantly different from the model-simulated networks (*p_combined_* > 0.05). We found that the main factor explaining the dominance interactions in the Floreana Mockingbird was age (Figure 1). Older individuals tended to interact aggressively with younger individuals (age homophily coefficient = −0.52 ± 1.6, *p* = 0.001; Table 1). Younger individuals were also less likely to initiate dominance interactions (actor effect of age coefficient = −0.27 ± 0.07, *p* = 0.0001; Table 1). We did not find a significant effect for reciprocity, sexual homophily, or body size. MCMC did not show any signs of model degeneracy (Supplementary Material). Furthermore, the simulations of the modelled network were similar to our observed network, showing good model fit (Table 2).

### 3.2. Removal Scenarios

**Figure 2:**
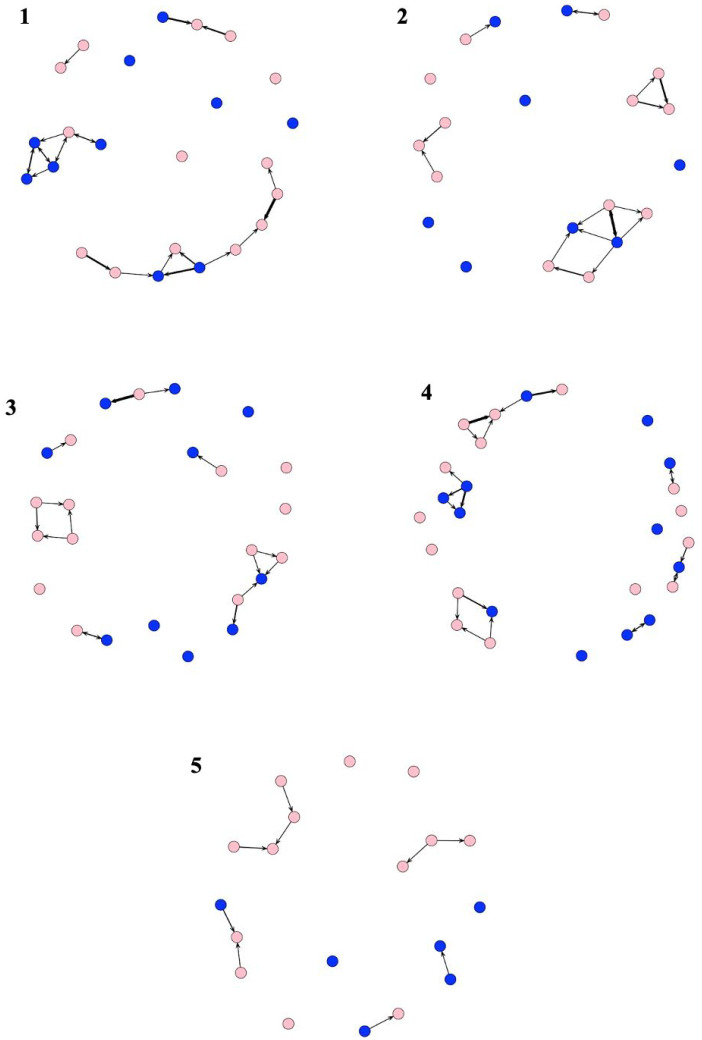
Simulated removal for translocations after the removal of 50% of individuals. Scenarios include: (1) removal of older individuals; (2) removal of juveniles; (3) removal of individuals with high betweenness; (4) removal of brokers; and (5) removal of randomly selected individuals. Nodes represent individual birds. Nodes in the network are displayed by colours representing sex; blue nodes are males and pink nodes are females.

The removal of individuals (*nodes*) with the strongest influence on the social network structure affected the number of components and isolates (Table 3, Figure 2). From the five simulations where disruption was expected, scenario 3 (removal of individuals with high betweenness centrality) and scenario 4 (removal of brokers) had a strong negative effect on the structure of the social network, resulting in more components and isolates compared with the observed network (Table 3).

## 4. Discussion

Despite our small sample size and limited duration of our observations, we show that Floreana Mockingbird family groups form transitive (linear) relationship structures. Our findings are comparable to other cooperative breeding species where transitivity methods have been tested [34,63]. This transitiveness was expected due to the difference in individual attributes that establish dominance ranks and resource accessibility, and self-organisation [64].

Of the four hypothesised drivers of network structure, only the two age-related factors explain dominance within Floreana Mockingbird family groups. Curry [6] previously proposed that age is the main predictor of social status in the Floreana and Galápagos Mockingbird (*M. parvulus*). In the Galápagos Mockingbird, older individuals display dominance relationships with younger individuals even within the same-age cohort (older juveniles are dominant toward younger individuals) [6]. Similarly, age is related to reproductive state and dominance in breeding groups of the facultative cooperative breeder White-breasted Thrasher (*Ramphocinclus brachyurus*) [3]. In Pied Babblers (*Turdoides bicolor*), older dispersing individuals are more likely to secure dominant breeding positions [65]. In Long-tailed tits (*Aegithalos caudatus*), age and size are related to dominance position within the group [66].

Considering the correlation between age and social status, we found that the probability of link formation through aggressive interactions increased when the social status decreased. Older individuals engage more in dominance with younger individuals than with other older individuals (differential homophily by age). Aggressive interactions were directed primarily from dominant to subordinate individuals, consistent with the expected structure of a strictly linear dominance hierarchy [50] and observed in Galápagos Mockingbirds [67], and other cooperative breeding systems, as a way of keeping the reproductive skew towards dominant individuals and subordinates in reproductive suppression [68,69,70,71]. The absence of an effect of reciprocity in our study is consistent with this observation. In contrast to our prediction, we found no effect of body size or sex on network structure. In some cooperative breeding species, phenotypic traits such as plumage coloration [72] and body size [66,73] are strong predictors of dominance. In other cooperative breeding systems, aggression by sexual homophily is explained by intrasexual competition for breeding positions within groups [34,63,74].

We found no effect of sexual homophily (aggression between same-sex dyads), in contrast to *M. parvulus*, where females are subordinate to males and female dominance over other females is associated with the rank of the female’s mate [6]. The dominance pattern we observed between sexes may be associated with the sex ratio of the population [47] and the use of a plural cooperative breeding strategy. Plural cooperative breeding occurs when more than one female reproduces within the family group or when multiple females share the same nest [75]. Floreana Mockingbirds rarely display plural breeding, unlike *M. parvulus* and *M. macdonaldi* [6,42]. The rarity of this strategy might reduce the frequency of intrasexual competition because, in non-plural breeding groups, all the members are closely related [43]. Finally, the absence of a sex effect could be because our observations were during the non-breeding season, when family groups are smaller (pers. obs.). For some social animals, the social network structure changes with reproductive stage [35,76].

Our simulations support the prediction that the removal of individuals would affect social dynamics and network stability. Similar effects have previously been shown in colonial bats (*Myotis septentrionalis*) using simulated removal of multiple roosting sites, resulting in network fragmentation increasing linearly with the proportion of roosting sites removed [77]. For the cooperative breeding fish *Astatotilapia burtoni*, the removal of dominant individuals results in social instability and competition for territories [13]. Our simulations also supported the hypothesis that strategic removal of individuals is required to minimise network disruption in the source population. Specifically, the removal of individuals with high betweenness and brokers (scenarios 3 and 4) increased the number of components and isolates of our networks. Brokers have been suggested as an important weakness in network structure and are usually targeted for removal when the purpose is the collapse of criminal social networks in humans [78,79]. Likewise, the removal of random individuals in a simulated social network of Killer Whales (*Orcinus orca*) was more stable than the removal of targeted individuals who simulated real-life captures [14]. Further, highly hierarchical networks are more prone to collapse because individuals may have specific roles within a network [11].

## 5. Conclusions

### Implications for Conservation and Limitation Acknowledgement

The lack of consideration of social structure in conservation practice may influence translocation outcomes in cooperative breeding birds, although its effect on overall success remains unclear. Translocations to date have generally focused on the movement of specific age classes, mainly juveniles, rather than moving entire groups (e.g., refs. [80,81]). A commonly observed outcome has been the disruption of the social system, with birds becoming social pair breeders without helpers during the early stages following release [15,16,17,18]. However, such social disruption does not necessarily mean translocation failure, as social systems can reorganise over time, and populations may still establish successfully.

To the best of our knowledge, only two cases of translocations of cooperative breeding birds have had no social disruption: first, the Brown Treecreeper, *Climacteris picumnus,* where entire family groups were translocated [23]; and second, Black-eared Miner (*Monarina melanotis*), a species with a plural breeding system [21].

The endangered Floreana Mockingbird is a target species for reintroduction to Floreana Island as part of the Floreana Island Restoration Project [82]. Based on our analyses, we suggest that translocating entire family groups may be a viable alternative to the current practice of annual transfer of individuals without regard to group membership [83]. Our findings are consistent with the hypothesis that cooperative breeders may be particularly sensitive to disruption of social networks and the removal of key individuals. Nonetheless, further research is needed to determine whether such disruption affects long-term establishment success, how social networks reorganise after translocations, and whether source populations can sustain the removal of intact family groups [39].

Given the endangered status of the Floreana Mockingbird, the limited number of remaining family groups, and logistical constraints associated with site accessibility, our study was restricted to three groups observed during the non-breeding season. Although these groups encompassed the range of age classes and social roles typically found in the population, further studies involving additional groups, seasons, and years are needed to assess the generality and temporal stability of the observed dominance patterns. Because age was the primary predictor of dominance, we expect the overall hierarchical structure to be relatively stable over time; however, changes in group composition and reproductive status may alter specific social relationships. Seasonal variation in social structure should also be considered when planning translocations, as non-breeding social relationships may be particularly relevant for maintaining group cohesion after release, whereas breeding-season dynamics may have greater implications for reproductive competition and long-term population establishment.

## Figures and Tables

**Table 1:** Terms and estimates from the ERGM fit of a Floreana Mockingbird dominance network. ERGM statistical terms from Box 2 have been “translated” to a simple terminology where Nonzero = non_zero, Actor effect of Sex = node_factor, Sexual homophily = node_match for sex, age homophily = node_match for age, Difference in body size = abs_diff, actor effect of age = node_ocov, receiver effect of age = node_icov and Reciprocity = mutual.

Model Term	Estimate	SE	*p* Value
Sum	1.24	0.24	**<0.0001**
Nonzero	−3.15	0.28	**<0.0001**
Actor effect of Sex (male)	−0.05	0.08	0.55
Sexual homophily	0.01	0.11	0.87
Age homophily	−0.52	0.16	**0.001**
Difference in body size	0.004	0.008	0.64
Actor effect of age	−0.27	0.07	**0.0001**
Receiver effect of age	0.01	0.03	0.58
Reciprocity	0.17	0.15	0.25

**Table 2:** Statistical parameters from the observed network and the mean statistical parameters of the model-simulated networks.

	Networks	
Parameters	Observed	Simulated
Sum	137	141.5
Nonzero	62	64.6
Actor effect of Sex (male)	114	126.5
Sexual homophily	67	76.2
Age homophily	35	38.5
Difference in body size	908.07	860.7
Actor effect of age	189	202.3
Receiver effect of age	429	479.6
Reciprocity	−119	−126

**Table 3:** Impact of node removals by different translocation simulations on the structure of the observed social network. Scenarios include: (1) removal of older individuals (birds > 1 year old), (2) removal of juveniles (birds < 1 year old), (3) removal of individuals with high betweenness, (4) removal of brokers, and (5) randomly selected individuals were moved. Scenarios such as numbers 3 and 4 with more components and isolates represent socially disrupted networks with more network subgroups and isolated individuals with no connection to other individuals in the network.

	Components	Isolates
Observed	4	0
Simulated Scenario 1	9	5
Scenario 2	10	5
Scenario 3	12	6
Scenario 4	13	7
Scenario 5	10	5

## Data Availability

Due to the privacy policy of the Galapagos National Park, the datasets used in preparing this paper are only available from the corresponding author on reasonable request.

## Supplementary Materials

The following supporting information can be downloaded at https://www.mdpi.com/article/10.3390/biology15120912/s1: Study area, Table of birds, Testing ERGM Model Fit, MCMC Diagnostic of the model and graphs.
