# Supplementary material for: Using Social Networks and Model Simulations of Social Disruption to Identify Alternative Translocation Strategies for the Endangered Cooperative-Breeding Floreana Mockingbird
Source: Biology (Basel). 2026 Jun 10;15(12):912. doi: 10.3390/biology15120912 (PMC13295389; doi:10.3390/biology15120912)
Supplement: Supplementary file 1 [file biology-15-00912-s001.zip › biology-4342165-supplementary.pdf]

Supplementary material A

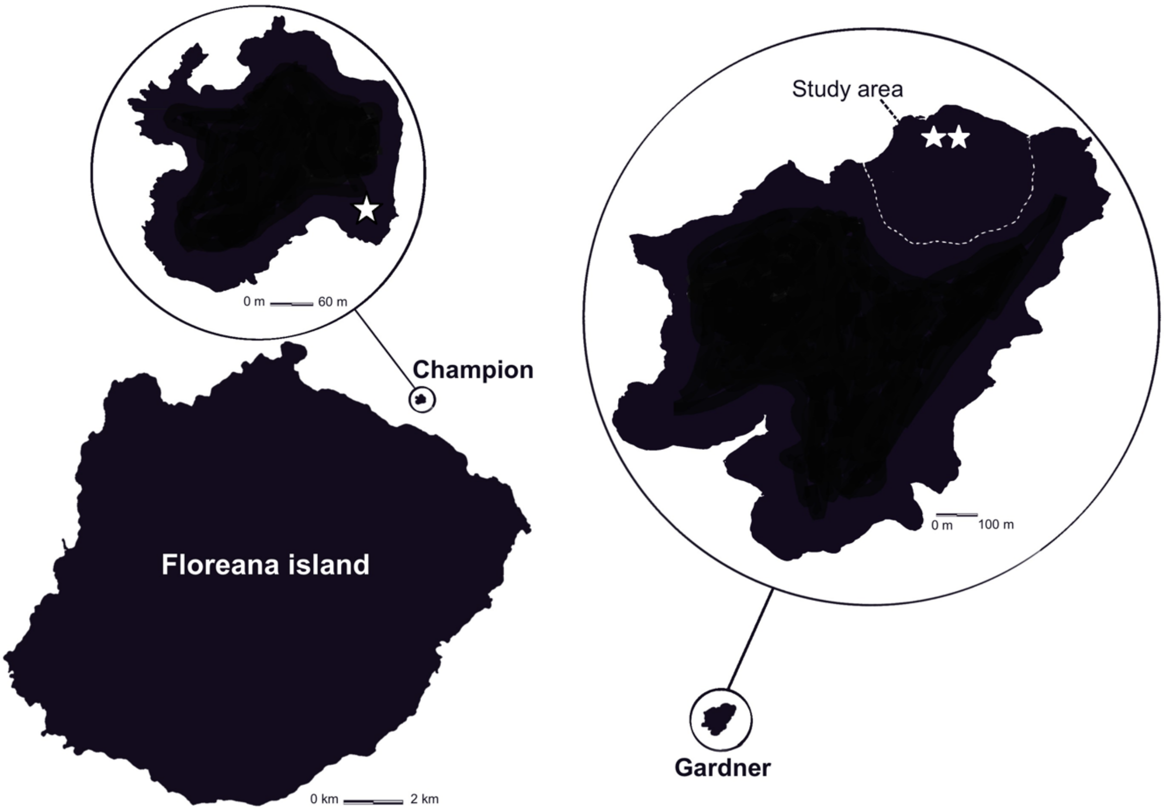

Supplementary Figure S1. Locations of the study groups of the Floreana Mockingbird (*Mimus trifasciatus*) on Champion Island and Gardner-by-Floreana Island. Stars indicate the approximate locations of the sampled family groups.

Supplementary material B

Supplementary Table S1. Group membership, sex, and age (as of 2019) of the Floreana Mockingbirds included in this study.

ID	Group	SEX	YEAR
C97082	A	M	1
C97083	A	F	1
C97121	A	F	1
S143477	A	F	7
S143545	A	F	4
S143578	A	M	4
S143793	A	M	4
S143795	A	M	4
C97080	A	F	1
C97009	A	F	3
C97085	A	F	1
S143794	A	M	4
C97087	B	F	1
C97088	B	F	1
C97038	B	M	2
C97086	B	F	1
C97089	B	F	1
C97065	B	F	1
C97183	B	F	1
C97111	B	M	1
S143470	B	M	7
C97001	B	F	1
C97035	B	M	2
C97185	B	M	1
C97098	B	M	1
C97099	B	M	1
S143711	C	F	5
S143710	C	M	5
C97059	C	F	2
C97128	C	M	1
S143623	C	M	9
C97138	C	F	1
C97126	C	F	1
C97127	C	F	1
C97125	C	M	1
C97136	C	F	1
S143783	C	F	4

Supplementary material C
Testing ERGM Model Fit

MCMC Diagnostic of the model and graphs
Sample statistics summary:

Iterations = 107172:2138292
Thinning interval = 624
Number of chains = 1
Sample size per chain = 3256

1. Empirical mean and standard deviation for each variable,
plus standard error of the mean:

	Mean	SD	Naive SE	Time-series SE
sum	0.494472	17.774	0.3115	0.9462
nonzero	0.228501	7.002	0.1227	0.2141
nodematch.sum.Sex	0.224816	12.740	0.2233	0.6528
nodematch.sum.Age	0.206695	8.628	0.1512	0.2834
absdiff.sum.SMI	-0.224340	168.533	2.9535	10.3387
nodefactor.sum.Sex.M	-0.009828	19.313	0.3385	1.0272
nodeicov.sum.Age	0.840295	28.670	0.5024	1.1675
nodeocov.sum.Age	-1.953624	70.587	1.2370	4.4195
mutual.nabsdiff	-0.100737	15.298	0.2681	0.7664

2. Quantiles for each variable:

	2.5%	25%	50%	75%	97.5%
sum	-32.00	-12.0	0.00	12.0	36.0
nonzero	-13.00	-4.0	0.00	5.0	15.0
nodematch.sum.Sex	-23.00	-9.0	0.00	9.0	26.0
nodematch.sum.Age	-16.00	-6.0	0.00	6.0	18.0
absdiff.sum.SMI	-295.19	-120.6	-13.69	106.1	363.6
nodefactor.sum.Sex.M	-36.00	-13.0	0.00	13.0	40.0
nodeicov.sum.Age	-51.00	-19.0	0.00	19.0	60.0
nodeocov.sum.Age	-130.00	-52.0	-5.00	44.0	147.0
mutual.nabsdiff	-31.62	-10.0	0.00	11.0	28.0

Are sample statistics significantly different from observed?

	diff.	test stat.	P-val.
sum	0.4944717	0.5225858	0.6012625
nonzero	0.2285012	1.0673428	0.2858171
nodematch.sum.Sex	0.2248157	0.3444004	0.7305452
nodematch.sum.Age	0.2066953	0.7292402	0.4658547
absdiff.sum.SMI	-0.2243397	-0.0216991	0.982688
nodefactor.sum.Sex.M	-0.009828	-0.0095682	0.99236582
nodeicov.sum.Age	0.8402948	0.7197408	0.4716846

nodeocov.sum.Age	-1.9536241	-0.4420433	0.6584579
mutual.nabsdiff	-0.1007371	-0.1314492	0.89542
(Omni)	NA	4.7545115	0.8596814

Sample statistics cross-correlations:

	sum	nonzero	nodematch.sum.Sex	nodematch.sum.Age	absdiff.sum.SMI	nodefactor.sum.Sex	nodeicov.sum.Age	nodeocov.sum.Age	mutual.nabsc
sum	1	0.8340385	0.6914849	0.4618423	0.7090045	0.7535034	0.8443459	0.7976784	-0.8843091
nonzero	0.8340385	1	0.5784025	0.5017261	0.5753065	0.6207132	0.7664012	0.6001417	-0.7027064
nodematch.sum.Sex	0.6914849	0.5784025	1	0.3611452	0.5689276	0.3941795	0.5864202	0.5303244	-0.6002924
nodematch.sum.Age	0.4618423	0.5017261	0.3611452	1	0.3151577	0.2634082	0.3134228	0.1206545	-0.4234337
absdiff.sum.SMI	0.7090045	0.5753065	0.5689276	0.3151577	1	0.4613213	0.6031323	0.5449043	-0.6263231
nodefactor.sum.Sex.M	0.7535034	0.6207132	0.3941795	0.2634082	0.4613213	1	0.630814	0.6594738	-0.6688921
nodeicov.sum.Age	0.8443459	0.7664012	0.5864202	0.3134228	0.6031323	0.630814	1	0.6242639	-0.6851048
nodeocov.sum.Age	0.7976784	0.6001417	0.5303244	0.1206545	0.5449043	0.6594738	0.6242639	1	-0.7893686
mutual.nabsdiff	-0.8843091	-0.7027064	-0.6002924	-0.4234337	-0.6263231	-0.6688921	-0.6851048	-0.7893686	1

Sample statistics auto-correlation:
Chain 1

	sum	nonzero	nodematch.s	nodematch.sum.Age	absdiff.sum.SMI	nodefactor.s	nodeicov.sum.Age	nodeocov.sum.Age	mutual.nabsdiff
Lag 0	1	1	1	1	1	1	1	1	1
Lag 624	0.6568956	0.32913319	0.672264	0.42694085	0.717773	0.6653432	0.4606934	0.7676973	0.6098512
Lag 1248	0.5000947	0.17318152	0.5289962	0.23659985	0.5791573	0.5167333	0.323209	0.6395367	0.4710567
Lag 1872	0.4081169	0.11618925	0.4345566	0.16476106	0.5022334	0.4213215	0.2507308	0.549513	0.3803076
Lag 2496	0.3509727	0.10008173	0.3726552	0.14130949	0.4549115	0.3609165	0.2084989	0.4726784	0.328423
Lag 3120	0.3055242	0.07262506	0.317239	0.09413953	0.4106696	0.3049822	0.1776432	0.4257403	0.2938176

Sample statistics burn-in diagnostic (Geweke):
Chain 1

Fraction in 1st window = 0.1
Fraction in 2nd window = 0.5

sum	nonzero	nodematch.sum.Sex	nodematch.sum.Age
0.01674827	-0.08638115	-0.58880932	1.57557390

absdiff.sum.SMI	nodefactor.sum.Sex.M	nodeicov.sum.Age	nodeocov.sum.Age
-0.38596028	-0.71916329	-1.05942443	0.89168143

mutual.nabsdiff
-0.27086492

Individual P-values (lower = worse):

sum	nonzero	nodematch.sum.Sex	nodematch.sum.Age
0.9866374	0.9311634	0.5559892	0.1151240

absdiff.sum.SMI	nodefactor.sum.Sex.M	nodeicov.sum.Age	nodeocov.sum.Age
0.6995261	0.4720403	0.2894065	0.3725637

mutual.nabsdiff
0.7864949

Joint P-value (lower = worse): 0.02846308

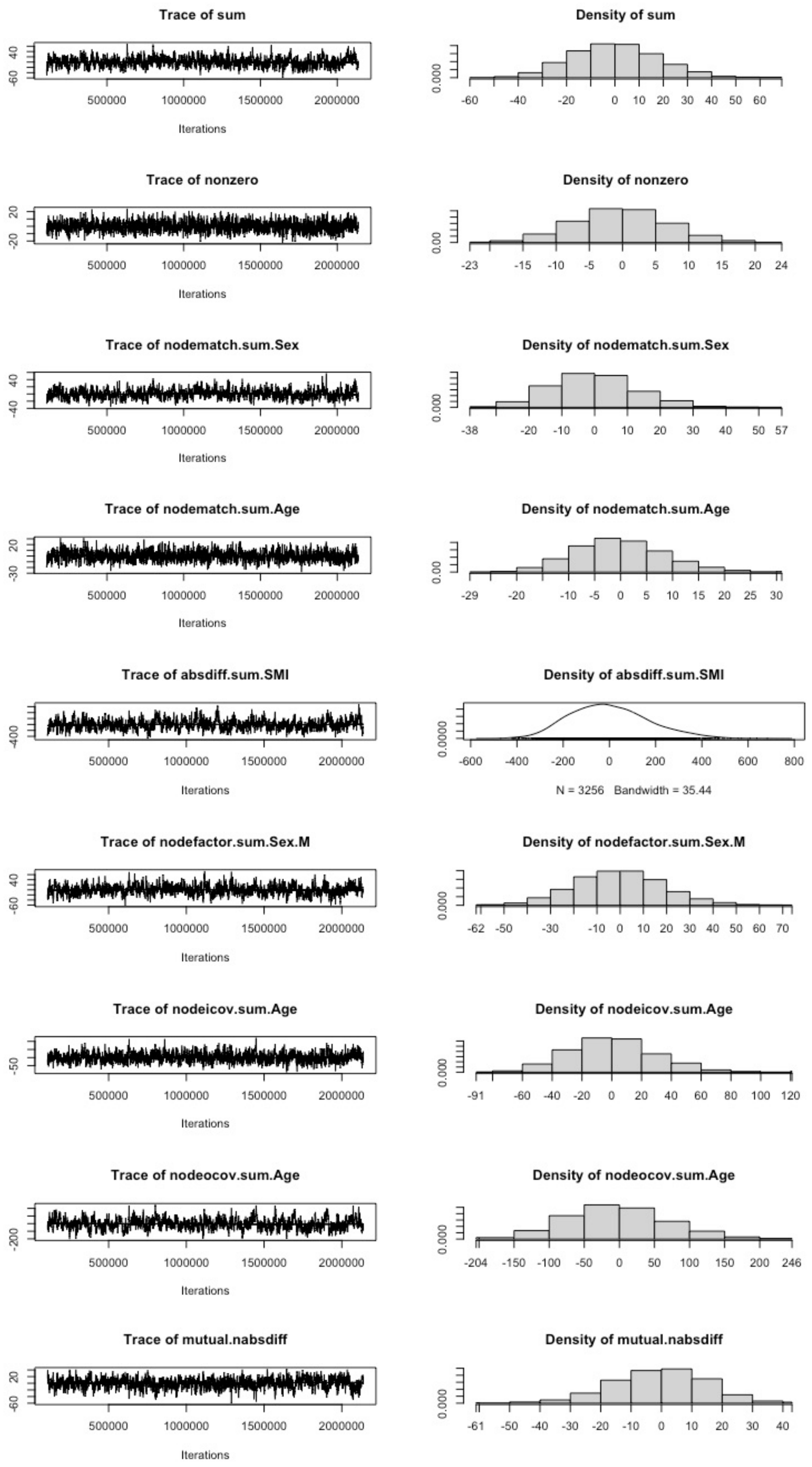
Trace of sum
Iterations
Density of sum
Trace of nonzero
Iterations
Density of nonzero
Trace of nodematch.sum.Sex
Iterations
Density of nodematch.sum.Sex
Trace of nodematch.sum.Age
Iterations
Density of nodematch.sum.Age
Trace of absdiff.sum.SMI
Iterations
Density of absdiff.sum.SMI
N = 3256 Bandwidth = 35.44
Trace of nodefactor.sum.Sex.M
Iterations
Density of nodefactor.sum.Sex.M
Trace of nodeicov.sum.Age
Iterations
Density of nodeicov.sum.Age
Trace of nodeocov.sum.Age
Iterations
Density of nodeocov.sum.Age
Trace of mutual.nabsdiff
Iterations
Density of mutual.nabsdiff
